# Algal Ocelloids and Plant Ocelli

**DOI:** 10.3390/plants12010061

**Published:** 2022-12-22

**Authors:** Felipe Yamashita, František Baluška

**Affiliations:** Institute of Cellular and Molecular Botany, University of Bonn, 53115 Bonn, Germany

**Keywords:** algae, cyanobacteria, eyes, eyespots, ocelloids, ocelli, plants, roots, shoots, vision

## Abstract

Vision is essential for most organisms, and it is highly variable across kingdoms and domains of life. The most known and understood form is animal and human vision based on eyes. Besides the wide diversity of animal eyes, some animals such as cuttlefish and cephalopods enjoy so-called dermal or skin vision. The most simple and ancient organ of vision is the cell itself and this rudimentary vision evolved in cyanobacteria. More complex are so-called ocelloids of dinoflagellates which are composed of endocellular organelles, acting as lens- and cornea/retina-like components. Although plants have almost never been included into the recent discussions on organismal vision, their plant-specific ocelli had already been proposed by Gottlieb Haberlandt already in 1905. Here, we discuss plant ocelli and their roles in plant-specific vision, both in the shoots and roots of plants. In contrast to leaf epidermis ocelli, which are distributed throughout leaf surface, the root apex ocelli are located at the root apex transition zone and serve the light-guided root navigation. We propose that the plant ocelli evolved from the algal ocelloids, are part of complex plant sensory systems and guide cognition-based plant behavior.

## 1. Introduction

Vision in animals is incredibly diverse and it evolved multiple times independently [1,2,3]. Despite a great diversity of visual organs, an eye can be defined as the existence of a cornea and/or lens which focuses the light towards a sensory region, such as eye retina or other light-sensitive structures and tissues, with photo-responsive proteins transforming the light signal first into electrical and then into chemical signals [4,5,6].

In 1905, Gottlieb Haberlandt proposed the plant ocelli concept for leaf epidermis in which the upper epidermal cells resemble convex or planoconvex lens, converging light rays on the light-sensitive subepidermal cells [7]. The Haberlandt plant ocelli theory is not surprising if we consider that various organisms such as bacteria, algae, and fungi (as discussed below) have cells with similar light-sensing properties. However, plant ocelli theory was almost forgotten and only recently revived [8,9]. Supporting this leaf epidermal ocelli scenario, leaf epidermis cells, with the exception of stomata guard cells, do not generate photosynthetic chloroplasts, although they have the best position with respect to the amount of light they receive.

This concept was recaptured some 70 years later when young seedlings of tropical vine *Monstera gigantea* were reported to localize and suitably support host trees using growth towards darkness termed *skototropism*—the directional movement of plant organ towards darkness [10]. Due to observations, and apart from other theories, Strong and Ray (1975) found skototropism to be the relevant mechanism in the finding of host trees by the Monstera vine. They provided evidence that shoot skototropism is an independent mechanism. Nevertheless, they assumed it to be a modification of negative phototropism. Additionally, they reported a negative effect of increasing distance and a positive effect of increasing host stem diameter on the shoot skototropism. Importantly, the larger a potential host tree is and the closer it is located to the vine seedling, the stronger the skototropic response will be [10].

## 2. Chlamydomonas Algal Eyespot: Rhizoplast and Rootlet Connections

**Figure 1:**
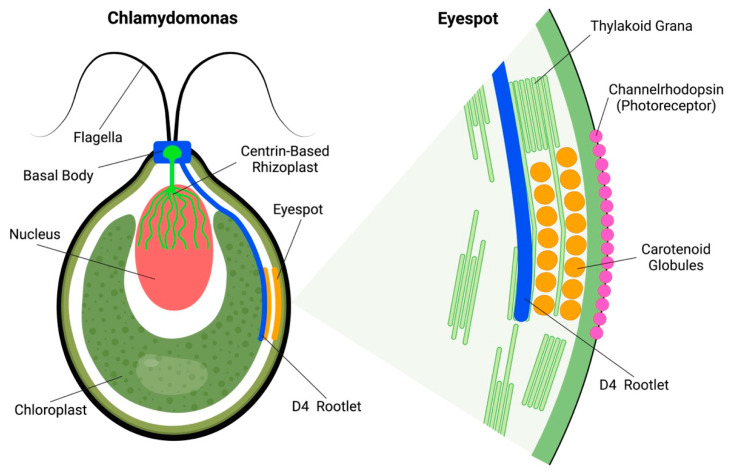
**Algal Eyespot of Chlamydomonas.** Chlamydomonas alga with two flagella associated with the basal bodies which intracellularly organize intracellular bundles of microtubules (known as rootlets) of which the D4 bundle anchors the eyespot. This eyespot is constructed from chloroplast thylakoid membranes and carotenoid globules, aligned under the plasma membrane which is enriched with photoreceptor channelrhodopsin. Besides the bundles of microtubules, the basal body also organizes the centrin-based contractile nucleo-basal body connector anchoring the nucleus. M4, M2 and D2 rootlets are not shown in this simplified scheme.

The green alga *Chlamydomonas reinhardtii* also has a subcellular eyespot apparatus. Algal eyespots are anchored at the Chlamydomonas cell periphery via so-called D4 bundles of microtubules, organized by the basal body (Figure 1). In addition, an important—but often neglected—feature of Chlamydomonas is the rhizoplast, which is a contractile centrin-based structure connecting basal bodies of flagella with the nuclear surface [11,12,13]. These so-called rhizoplasts or fibrous flagellar roots anchor nuclei to the flagellar or ciliar basal bodies [14,15,16,17,18,19]. The eyespot of Chlamydomonas is anchored to the D4 rootlet, extending from the peripheral flagellar basal bodies into the cell interior [20,21,22]. Intriguingly, similarly to the scenario with the ocelloids of the warnowiid dinoflagellates discussed below, these algal eyespots are also assembled from putatively symbiotic components. Besides the chloroplasts, there is cellular evidence suggesting that the nucleus–basal body–flagellum/cilium complex is of symbiotic origin, representing the guest cell of the host–guest cell consortium [23,24].

*Chlamydomonas* green algae have two vision responses. The first one is swimming in towards or away from light ray source, called phototaxis, depending on the total amount of reactive oxygen species (ROS) inside the cell [25,26]. The second is when they freeze for a few moments after receiving a strong light stimulus, followed by a backstroke, and then swimming normally in any direction. This second one is called photo-shock response: as the name implies, the algae stop their natural movement for seconds [27,28]. Under a microscope, it is easy to find the eyespots, as they are composed of orange carotenoid globules located under the plasma membrane enriched with photoreceptor proteins, channelrhodopsinsChR1 and ChR2 [29]. In green alga *Chlamydomonas reinhardtii*, the eyespot apparatus is composed of two layers of carotenoid globules (Figure 1) sandwiched between the thylakoid membranes of the chloroplast [28,30,31]. The eyespot apparatus is activated through light stimuli, and afterwards controls flagella to accomplish phototaxic behavior [30]. An important aspect is that the light-induced eyespot electric currents activate and control the flagellar currents via the electric action potential-like transmission [32,33,34,35]. Rapid calcium influxes and bioelectric currents integrate sensory events at the eyespot with control of flagella beating and phototaxis [27,32,33,36].

Another algae protist that evolved a light-sensitive apparatus adapted for unicellular vision is *Euglena gracilis*. It shows two basic types of photo-movements in response to light stimuli, known as photophobic and phototactic behaviors. Similarly, as in the eyespot of Chlamydomonas, *Euglena gracilis* carotenoids are important for photo-movements. The plastids do not develop into chloroplasts due to the lack of chlorophyll synthesis [37,38]. Recent studies have reported that mutants, deficient in carotenoid production, lose their phototactic responsiveness [38]. Carotenoids are obviously essential for light perception of the Euglena eyespot. Similarly, as in Chlamydomonas, the eyespot of Euglena is associated with the microtubules-based flagella [37,39,40]. However, *Euglena gracilis* obtained their plastids much later via the secondary endosymbiosis and are evolutionary distant, belonging to Archaezoans [41]. Thus, it is not surprising that Euglena and Chlamydomonas rely on different photoreceptors in their ocelli.

## 3. From Algal Ocelloids to Plant Ocelli

**Figure 2:**
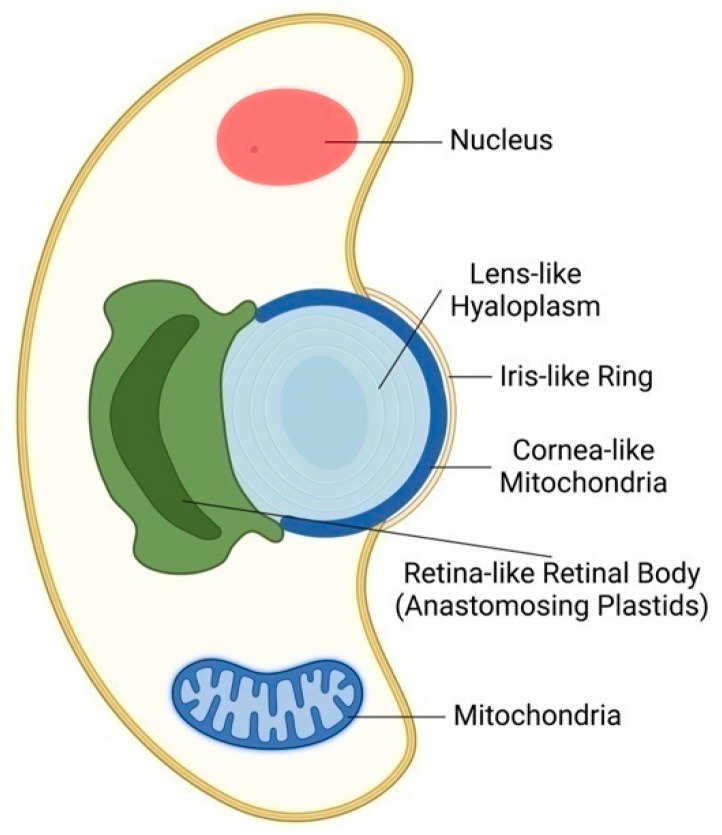
**Algal Ocelloid of Dinoflagellates.** Camera-like ocelloid of warnowiid dinoflagellates is composed of cornea-like mitochondrion enclosing hyaloplasm acting as lens and chloroplast-based retinal body. Similarly, as in the algal eyespot, the chloroplast plays the central role in the microbial vision. Adapted according [43].

In 1967, David Francis described an eyespot in *Nematodinium armatum*, describing lenses capable of focusing light rays and concentrating them into a structure called a pigment cup. This structure is supposed to be a light-sensitive retinoid and may have a role in image formation [42]. In 2015, further unexpected support for the plant ocelli theory of Gottlieb Haberlandt was provided with the surprising discovery of eye-like ocelloids in warnowiid dinoflagellates [43,44]. These planktonic unicellular organisms use symbiotic organelles which act as eye-like ocelloids. A mitochondria-based layer generates a cornea-like surface across a lens structure, whereas the retinal body of ocelloids develops from a membrane network formed from plastids (Figure 2). To verify these microscopically based findings, the scientists sequenced the DNA of a warnowiid retinal body, which had a substantially greater percentage of DNA originating from plastids than comparable samples from the total cell [43]. Warnowiid dinoflagellates are the only unicellular microbial organism having camera-type eye-like organs for camera-type vision-like modus [4,42,43,44,45].

## 4. Bacterial Vision: Cyanobacterium Synechocystis

**Figure 3:**
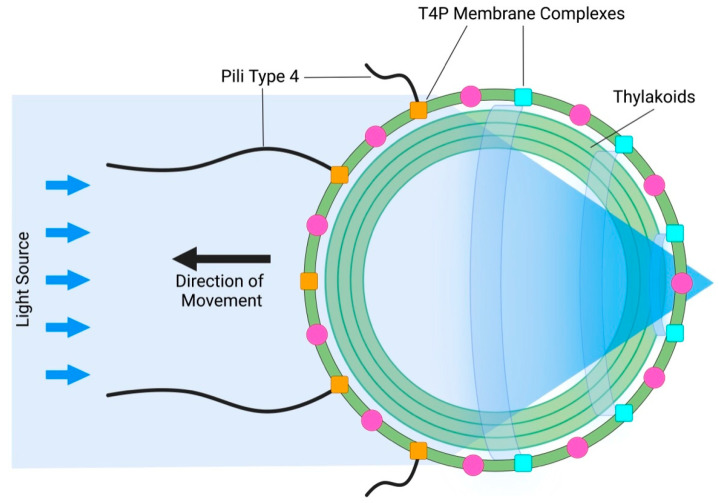
**Bacterial Vision: Cyanobacterium Synechocystis.** The whole cyanobacterial cell acts as a lens, focusing light beams on a small patch of the plasma membrane which controls the type-IV pili-based motility apparatus anchored in the plasma membrane via T4P complexes. Under the plasma membrane are thylakoid membranes. This model was adapted according to [49].

The next surprising discovery followed one year later, when Schuergers et al. (2016) reported prokaryotic bacterial vision in cyanobacterium Synechocystis sp. PCC 6803 [46,47,48,49]. Here, the whole cell acts as a lens, focusing light on a small patch of the plasma membrane (Figure 3). A similar principle, in which the whole cell acts as a lens, was found also in eukaryotic volvocine algae [50]. Therefore, it should not be surprising if plant cells also rely on this feature via their ocelli. Importantly, biological evolution repeatedly uses all the successfully elaborated structures and processes which improve the organismal survival chances. Even the most complex organs of vision, such as animal and human eyes, represent the inherent part of the long evolutionary continuum. In the case multi-cellular volvocine algae, light-focusing roles of cells affect the adjacent cells in a manner which participates in morphological symmetries and colony behavior as relevant information [50]. In Synechocystis, light perception at the photosensitive patch of the plasma membrane electrically controls type IV pili-based motility apparatus [51] in such a manner that pili close to the light focal spot are inactivated, whereas pili on the opposite side of the cell (facing the light source) are active and allow movement towards the light source [46,47,48,49]. As cyanobacteria evolved more than three billion years ago, it is obvious that this ancient prokaryotic vision based on the type IV pili complex is a very successful solution to their environmental challenges [52,53].

## 5. Plant Vision: *Boquila trifoliata*

Another example of an organism that can change its structures is the interesting plant *Boquila trifoliolata*, which can change its original three-lobed leaf shape into longitudinal leaves or any other shape, depending on the host plant next to its leaves. This is what the experiment by White & Yamashita (2021) illustrated [54]. The Boquila leaves were placed next to plastic leaves of non-living host plant, and the surprising result was that the Boquila mimicked the plastic plant leaves by changing leaf shape to a longitudinal shape, mimicking the plastic leaves of the non-living model plant. This experiment refutes two hypotheses proposed by other researchers. The first hypothesis was that horizontal gene transfer is mediated by the airborne microbes involved, thus allowing the Boquila to modify its leaves according to the leaves of the host plant. The second hypothesis was that the Boquila modified its leaves following some volatile chemical signals released by the host plant. As the plastic leaves of non-living host plants were able to induce mimicking response in the Boquila, the hypothesis of horizontal gene transfer and the hypothesis of volatile substances can be dismissed. The plastic leaves might release some volatile substance under sunlight exposure, but these are biologically not relevant. This is very strong support for the proposal that plant-specific vision based on leaf ocelli is behind the mimicking responses of Boquila plants. This would also explain that the Boquila leaves can actively identify their surrounding environment, and modify not only leaf sizes and forms, but also color, leaf vein networks and other anatomical patterns. Future experimental research is needed to understand how all this can be accomplished.

## 6. Root Apex Vision: Root Skototropism

**Figure 4:**
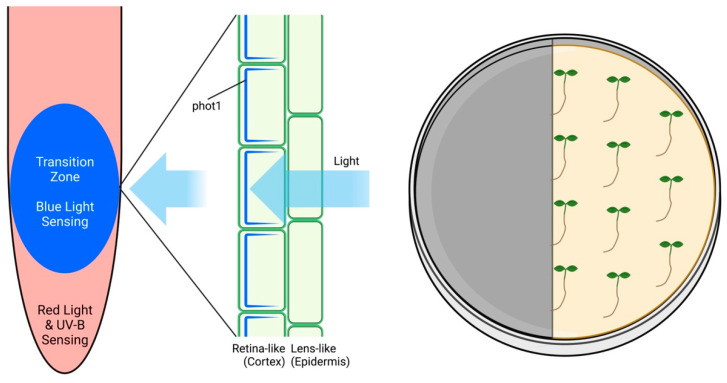
**Root Apex Ocelli.** Arabidopsis root apex expresses phot1 blue-light photoreceptor in cortex cells of the transition zone. The phot1 photoreceptors are arranged in the U-shape arrangements under the root epidermis cells which are devoid of phot 1 and are proposed to act as a lens cells, focusing the light on the underlying cortex cells. The root apex ocelli are proposed to allow root skototropism when roots grown within the illuminated portion of Petri dish can recognize the dark area and navigate the root growth towards it.

Although all roots of plants growing out in the nature are underground in darkness, they express all photoreceptors at their root apices [55]. While a dim light is not stressful for roots, they try to escape from stronger lights, which represent a stress factor for roots [56,57,58]. In order to avoid the direct illumination of roots in young seedlings grown in laboratory conditions using transparent Petri dishes, we have proposed the use of partially darkened dishes which allow us to keep roots in darkness [59,60,61]. Alternatively, the D-Root system was established as an alternative method to maintain roots in the shaded environment [62,63,64]. Surprisingly, roots grew even faster when grown within the D-Root system and our analysis revealed that this was due to steep light–darkness gradient provided by the D-Root system, which roots evaluate as a potent growth stimulant [65,66]. The process of speeding up the root growth under the steep light–darkness gradient of the D-Root system is based on the TOR complex activity, as its specific inhibition blocked this light escape tropism of illuminated roots [66]. Interestingly, roots placed in the illuminated portion of the shaded Petri dishes could recognize the dark portions of dishes, even when placed up to 2 mm from the light/darkness border (Figure 4). This implies some kind of root apex vision in the root apex skototropism response. The root apex ocelli proposed for this root skototropism are based on the blue-light phot 1 photoreceptor [55]. In contrast to diffusely distributed leaf epidermis ocelli, the root apex ocelli are assembled locally [67,68] at the root apex transition zone [69,70]. This position is optimal for the root apex vision, guiding the root apex navigation towards darkness [71].

## 7. Conclusions and Perspectives

**Figure 5:**
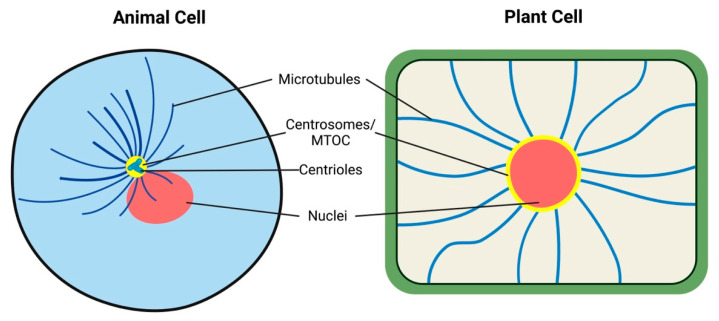
**Microtubules-MTOC in Rudimentary Cell Vision of Eukaryotic Cells.** Albrecht-Beuhler’s rudimentary cellular vision is accomplished via microtubules conveying infrared wavelengths along microtubules towards the perinuclear centrosome of animal cells. In the plant cells, the centrosome is not corpuscular but is distributed diffusely along the whole nuclear surface.

Vision via the whole organismal surface is known from some animals, such as cuttlefish and cephalopods [72,73]. Similarly, sea urchins and brittle stars have dispersed visual systems [74,75], all resembling the situation in plant leaf ocelli. Other lower animals have local eyes which resemble rather the root apex ocelli. Starfish have compound eyes at the arm tips [76,77]. Cnidarian medusae have eyes at the bases of their tentacles or on special sensory structures (rhopalia) which contain two lens-eyes flanked by two pairs of lens-less eyes [78]. Recent genetic studies have shown that the genes *Pax6*, *six1* and *six3* play key roles in the development of the eye in organisms from planaria to humans, arguing strongly for a monophyletic origin of the animal eye [79]. Nevertheless, there is no single regulatory gene in the formation of all animals. Diversity of vision in different animals must be based on gene expression as a tool and include the function of critical genes as mechanisms of the visual organ formation [79]. The hypothesis of phytochrome gene transfer from cyanobacteria, generating the first plastid in eukaryotes, paves the way for the presence of carotenoids in algae, which in turn are of extreme importance in eyespots [80]. Obviously, the leaf ocelli of plants conform well with algae and animal visual systems and represent obvious examples of convergent evolution. Root apex ocelli, based on the phot1 blue-light photoreceptor, represent another solution for the plant vision. It can be speculated that every cell with chloroplast has a cellular vision, resembling cells of cyanobacteria, algae, and plants. Albrecht-Buehler proposed 30 years ago that animal cells enjoy rudimentary vision [81,82,83,84,85] because they sense infrared wavelengths via their microtubules (Figure 5). This cellular vision is based on radial microtubules converging at their organizing centers (MTOCs), including centrosomes, basal bodies of cilia, and nuclear surfaces [86,87]. In future, it will be interesting to investigate the possible roles of microtubules in algal ocelloids and eyespots, as well as in plant leaves and root ocelli.

In conclusion, it emerges that vision is an ancient sensory faculty which evolved some three billion years ago with the very first cyanobacteria. Evolution never discards successful innovations, and the algal and plant vision is based on that of chloroplasts too.

## Data Availability

Not applicable.
